# Glenosphere Size Does Not Matter in Reverse Total Shoulder Arthroplasty

**DOI:** 10.1055/s-0043-1770976

**Published:** 2024-04-10

**Authors:** Akshar V. Patel, Christopher A. White, Troy Li, Bradford O. Parsons, Evan L. Flatow, Paul J. Cagle

**Affiliations:** 1 Departamento de Cirurgia Ortopédica, Icahn School of Medicine at Mount Sinai, New York City, New York, Estados Unidos

**Keywords:** arthroplasty, shoulder, scapula, shoulder joint, range of motion articular

## Abstract

**Objective** There are few studies to date reporting on outcomes following reverse total shoulder arthroplasty with cohorts stratified by glenosphere size. The purpose of this study is to investigate the role that glenosphere size has on postoperative outcomes.

**Methods** Patients who underwent reverse TSA between 1987 with minimum of 2.0 years of follow-up were included. Patients were stratified into two cohorts based on glenosphere size of 36mm or 40mm. Patients' range of motion, patient-reported outcomes, and radiographic variables (glenoid preoperative morphology, scapular notching, humeral loosening) were evaluated.

**Results** All measurements of range of motion measurements with the exception of internal rotation saw significant preoperative to postoperative improvements within each cohort. There were no significant differences in postoperative range of motion, ASES, or VAS pain scores across the two cohorts. Overall, forward elevation improved to 134° ± 16° in the 36mm cohort and 133° ± 14° in the 40mm cohort ( *p* = 0.47). External rotation improved to 37° ± 13° for 36mm patients and 35° ± 19° for 40mm patients ( *p* = 0.58). In the 36mm group, internal rotation increased by 1.3 vertebral levels and 2.3 vertebral levels in the 40mm cohort. At final follow-up, the 36mm cohort had a VAS score of 2 ± 2, ASES score of 66 ± 19, and SST score of 6 ± 3. Similarly, the 40mm cohort had a VAS score of 2 ± 3, ASES score of 77 ± 28, and SST score of 9 ± 3.

**Conclusions** Reverse TSA provides sustained improvements in range of motion and shoulder function irrespective of glenosphere size.

**Level of Evidence** III.

## Introduction

Reverse total shoulder arthroplasty (reverse TSA) is an effective treatment for several shoulder arthropathies and has been shown to provide substantial improvements in pain and shoulder function. 1 2 3 In the past decade, there has been over a 3-fold increase in the number of reverse TSA procedures performed annually in the United States as the number of approved indications rapidly expanded. 4 5 6 7 8 9 10 11 12 13 While reverse TSA overall is clinically effective, questions remain on how different implant types and sizes affect outcomes. Specifically, the choice of glenosphere size is one of the few variables controllable by the operating surgeon.

There are conflicting reports about the impact that glenosphere size has on functional outcomes following reverse TSA. Mollon et al. 14 and others suggest that larger glenospheres may correlate with better functional outcomes and improved radiographic results. 15 16 17 On the other hand, Schoch et al. and others found insufficient evidence to support the claim that glenosphere size significantly affects functional outcomes. 18 19 Sabesan et al. 20 also report that any difference in functional outcomes is hard to attribute to any one variable as it is difficult to control for demographic and anatomic variations in the patient population, specifically with regards to consistent placement of the glenosphere. Additionally, while the choice of glenosphere size is one of the few variables controllable by the operating surgeon, there are no established guidelines for determining the optimal size for each patient. It is commonly known that glenosphere size usually comes down to surgeon preference as a smaller glenosphere lends itself to easier insertion but a larger glenosphere may reduce the risk of future dislocation.

The purpose of this study is to contribute to the existing literature and evaluate the impact that glenosphere size has on functional outcomes following reverse TSA at mid- to long-term follow-up. We hypothesized that there would be no significant difference between glenosphere sizes with regards to shoulder function and pain.

## Methods

### Patient Population

The institutional review board approved this study. The institutional shoulder arthroplasty registry was queried using CPT code 23472 to identify the patients who had undergone a reverse total shoulder arthroplasty. Each chart was screened, and patients were included if they had a minimum follow-up of 2.0 years. Patients were excluded if they had an anatomic TSA, hemiarthroplasty, or follow-up time less than 2.0 years. Demographic information such as age at surgery, sex, revision status, and BMI were recorded. Implant survival was defined as shoulders that did not go onto revision following index reverse TSA. Implant size was determined intra-operatively based on anatomic evaluation. Preoperative planning software was not utilized to determine the size before surgery.

### Clinical Evaluation

The clinical endpoints measured in this study were range of motion scores and patient reported outcomes. Range of motion scores include forward elevation, external rotation, and internal rotation values of the shoulder as evaluated by the operating surgeon at both preoperative and latest postoperative visits. Patient reported outcomes include self assessment scores comprising Visual Analog Scale (VAS), ASES, the Simple Shoulder Test (SST) scores, and conversion metrics. Internal rotation was categorized as defined by Amroodi et al. 21 Implant survival time in subjects, defining failure as either implant revision or removal, was conducted using Kaplan-Meier survival analysis. Any differences in survival distributions were determined using a log rank test.

### Radiographic Assessment

Radiographic analysis was conducted by two fellowship trained orthopedic surgeons (B.O.P and P.J.C). A fellow (S.M.) was available to review any discrepancies between the two reviewers. The preoperative glenoid morphology was analyzed using the Walch classification system. 22 Humeral lucency and glenoid loosening were defined using the system outlined in Sanchez et al. 23 ; radiolucent lines >2mm were recorded by zone. Scapular notching was also assessed.

### Statistical Analysis

Categorical variables were analyzed using either χ2 or Fisher's Exact test. Normality of continuous variables was determined by a Kolmogorov-Smirnov test from which a Mann Whitney U or student's *t* -test was run. Outcomes were expressed as mean ± standard deviation. Preoperative, postoperative, and change in pre- to post-operative range of motion (ROM), patient reported outcomes (PROs), and radiographic measures were compared using a one-way analysis of variance (ANOVA) test. A Pearson's coefficient was used to compare the association between overall BMI and patient's age at surgery, ROM, and PRO scores. A p-value < 0.05 was considered significant.

## Results

### Overall

50 shoulders met inclusion criteria and were included in this study. The glenosphere distribution was 36mm ( *n* = 38) and 40mm ( *n* = 12). 34/38 of the 36mm glenosphere patients were female, while only 2/12 of the 40mm cohort was female ( *p* < 0.01). There were 14 males and 36 females included in the study. There were 40 index rTSA cases and 10 revision rTSAs.

### 36mm Glenosphere Cohort

The average age at surgery was 72.1 ± 7.4 years with an average follow-up time of 6.7 ± 3.3 years. 34/38 patients were female (89%) and 4/38 were female (11%). Patients presented for surgery with an average BMI of 28.4 ± 5.9 with an average ASA score of 2.3 ± 0.6. The three most common indications for surgery were arthropathy ( *n* = 16), failed hemiarthroplasty for fracture ( *n* = 10), and chronic fracture dislocation with rotator cuff tear ( *N* = 3). Reverse TSA with latissimus dorsi tendon transfer was performed in 13 shoulders.

### 40mm Glenosphere Cohort

The average age at surgery was 74.1 ± 7.2 years with an average follow-up time of 4.8 ± 3.1 years. 10/12 patients were male. Patients presented for surgery with an average BMI of 27.3 ± 4.5 with an average ASA score of 2.5 ± 0.5. The three most common indications for surgery were arthropathy ( *n* = 6), failed hemiarthroplasty for fracture ( *n* = 4), and chronic fracture dislocation with rotator cuff tear ( *N* = 2). Reverse TSA with latissimus dorsi tendon transfer was performed in 2 shoulders.

### Outcomes: 36mm Cohort

All measurements of range of motion saw significant preoperative to postoperative improvements. Overall, forward elevation improved from 81° ± 46° preoperatively to 134° ± 16° postoperatively ( *p* < 0.01). External rotation improved from 26° ± 31° preoperatively to 37° ± 13° ( *p* < 0.01) while internal rotation improved from 1.3 vertebral levels ( *p* = 0.35). Significant improvements were also seen for each patient reported outcome index. ASES scores improved from 33 ± 16 preoperatively to 66 ± 19 postoperatively ( *p* < 0.01). SST scores improved from 2 ± 2 preoperatively to 6 ± 3 ( *p* < 0.01). VAS pain index scores went from a mean preoperative score of 6 ± 3 to a mean postoperative score of 2 ± 2 ( *p* < 0.01) ( Table 1 ).

**Table 1:** Comparison of postoperative outcomes

Measure	36mm	40mm	p-value
Forward Elevation ( ^o^ )	134 ± 16	133 ± 14	0.47
External Rotation ( ^o^ )	37 ± 13	35 ± 19	0.58
Internal Rotation	1.3	2.3	0.84
ASES	66 ± 19	77 ± 28	0.05
SST	6 ± 3	9 ± 3	<0.01
VAS	2 ± 2	2 ± 3	0.68

### Outcomes: 40mm Cohort

All measurements of range of motion saw significant preoperative to postoperative improvements. Overall, forward elevation improved from 74° ± 7° preoperatively to 133° ± 29° postoperatively ( *p* = 0.02). External rotation improved from 11° ± 28° preoperatively to 35° ± 19° ( *p* = 0.04) while internal rotation improved on average by 2.3 vertebral levels (p-value = 0.28). Significant improvements were also seen for each patient reported outcome index. ASES scores improved from 30 ± 19 preoperatively to 77 ± 28 postoperatively ( *p* < 0.01). SST scores improved from 3 ± 4 preoperatively to 9 ± 3 ( *p* < 0.01). VAS pain index scores went from a mean preoperative score of 7 ± 3 to a mean postoperative score of 2 ± 3 ( *p* < 0.01) ( Table 1 ).

### Comparison of Cohort Outcomes

There was no significant difference in range of motion across the two cohorts. For patient-reported outcomes, the 40mm cohort had a significantly higher SST score, but there was no difference in ASES or VAS scores. The 36mm cohort had four surgically revised complications while the 40mm cohort had none. For a complete comparison of postoperative outcomes, please see Table 1 .

### Radiography

Preoperative radiography was available for 30 shoulders. Walch glenoid classification was able to be done in eighteen 36mm shoulders: A1 ( *n* = 12), A2 ( *n* = 3), B2 ( *n* = 1), B3 ( *n* = 1), and D ( *n* = 1) and twelve 40mm shoulders: A1 ( *n* = 4), A2 ( *n* = 3), B2 ( *n* = 3), B3 ( *n* = 1), and D ( *n* = 1). The pre-RTSA morphology could not be assessed in three shoulders due to anterior glenoid fracture ( *n* = 2) and previous anatomic TSA ( *n* = 1). Postoperative radiography was available for all patients. Postoperative scapular notching was seen in 8/38 of the 36mm patients and 2/12 of the 40mm cohort at final follow-up (p-value = 0.46). Glenoid loosening was seen in three 36mm cohort shoulders. Humeral loosening was seen in six 36mm and three 40mm shoulders. Tuberosity resorption was seen in six 36mm glenosphere shoulders.

### Complications and Revisions

There were four complications that were surgically revised in the 36mm cohort. One index rTSA patient was revised two months following surgery for a disassembled glenosphere. One index rTSA patient experienced baseplate loosening 5.8 years following surgery, which was revised. One index rTSA patient was revised 8.9 years after surgery due to an infection. One revision rTSA patient had the prosthesis explanted due to an infection 3.8 years after reverse TSA. The 40mm cohort had no complications or revisions.

## Discussion

There have been several studies to date that investigate the role of glenosphere size on outcomes following reverse total shoulder arthroplasty. 14 16 17 24 While there is some literature to suggest that larger glenosphere sizes are associated with improved outcomes, other studies suggest there are no advantages to such an approach. In this study, we found that there was no significant difference in range of motion or patient-reported outcomes for patients who received a 36mm or 40mm glenosphere.

Earlier studies have suggested that a larger glenosphere size is associated with increased postoperative external rotation. 14 15 16 Haidamous et al found that patients with a larger glenosphere were significantly more likely to have an external rotation greater than 30° 16 Similarly, Mueller et al demonstrated that at 5 years follow-up, patients with a 44mm glenosphere had an average of 12° more external rotation than their 36mm counterparts. 14 A biomechanical cadaveric study by Langohr et al. 19 also demonstrated that increasing glenosphere size did not lead to an increased external rotation. Thus, there are conflicting findings surrounding the relationship between glenosphere size and external rotation. In our study, we found there was no significant difference in postoperative external rotation between the 36mm and 40mm glenospheres (36mm: 37°; 40mm: 35°; p-value = 0.58). However, our findings may be due to a much smaller sample size of 40mm glenospheres. We hypothesize that if there was an increased number of 40mm patients, that they would have a significantly higher external rotation. In a future study, it would be valuable to control for confounding variables such as patient age, activity level, and indication for surgery when assessing for the relationship between glenosphere size and external rotation.

Patient reported outcome scores are valuable in assessing self-reported shoulder function after reverse TSA. In a study investigating 370 38mm and 219 42mm glenospheres, Schoch et al found no significant difference in ASES and Constant scores at a mean follow-up of 2.6 years. 18 Similarly, Sabesan et al. 20 also demonstrated that a larger glenosphere size does not confer an advantage in regards to patient-reported outcomes. In our study, we found there were no significant differences in ASES or SST scores between the 36mm and 40mm cohorts at an average of 5–7 years following surgery. However, there was a significant difference in SST scores (36mm: 6 ± 3; 40mm; 9 ± 3; p-value < 0.01). Most notably, both cohorts reported an identical mean VAS score of 2, which suggests that all sizes of glenospheres are effective at reducing pain in reverse TSA candidates. These findings are promising - regardless of glenosphere size, patients can achieve excellent outcomes following reverse TSA.

There have been multiple studies to date investigating the role of glenosphere size on scapular notching in reverse TSA. 14 17 24 While there are some studies suggesting that a larger glenosphere reduces the risk of scapular notching, other studies suggest no such advantage exists. 14 15 17 24 In a mid-term follow-up study, Mueller et al found no significant differences in the incidence of scapular notching in patients who received a 36mm or 44mm glenosphere. 14 Similarly, Mollon et al. 17 found 10% of 38mm cohort and 9.5% of 42mm cohort had scapular notching at final follow-up. In a randomized controlled trial, Torrens et al observed that 49% of 38mm glenosphere patients had scapular notching while only 12% of 42mm patients had it. 24 Consistent with the previous retrospective studies, we found there was no significant difference in scapular notching based on glenosphere size. 21% of the 38mm and 17% of the 40mm cohort had scapular notching at final follow-up ( *p* = 0.49). However, further prospective research need to be conducted before definitive conclusions can be drawn regarding the role of glenosphere size in scapular notching. The studies suggesting no difference exists have been larger in size, but also retrospective in comparison to the smaller, but prospective randomized trial which found that a smaller glenosphere increases the risk of scapular notching.

There are limitations to consider with this study. The small number of 40mm patients made it difficult to draw definitive conclusions between the two cohorts. The lack of preoperative imaging for some patients reduces the ability to understand preoperative characteristics of patients in this study. The retrospective nature of this study creates an inherent bias in the results. Some patients who had undergone reverse TSA at our institution were deceased or lost to follow-up, which may have led to changes in the results.

## Conclusion

This study contributes to the literature investigating the relationship between glenosphere size and clinical outcomes. We found there were no significant differences in range of motion, patient-reported outcomes, or scapular notching between the 36mm and 40mm cohorts. Both glenosphere sizes provided long-term pain relief to patients. Importantly, there were no complications or revisions when a larger 40mm glenosphere was used.
