# Supplementary material for: Glenosphere Size Does Not Matter in Reverse Total Shoulder Arthroplasty
Source: Rev Bras Ortop (Sao Paulo). 2024 Apr 10;59(2):e254–9. doi: 10.1055/s-0043-1770976 (PMC11006517; doi:10.1055/s-0043-1770976)
Supplement: Related texts

# Research article: O tamanho da glenosfera não importa na artroplastia total reversa de ombro

**Keywords:** artroplastia, ombro, escápula, articulação do ombro, amplitude de movimento articular

## Abstract

**Objetivo** Até agora, poucos estudos relataram os desfechos da artroplastia total reversa (ATR) de ombro com coortes estratificadas pelo tamanho da glenosfera. O objetivo deste estudo é investigar o papel do tamanho da glenosfera nos desfechos pós-operatórios.

**Métodos** O estudo incluiu pacientes submetidos à ATR de ombro desde 1987 com acompanhamento mínimo de 2,0 anos. Os pacientes foram estratificados em duas coortes com base no tamanho da glenosfera (36 mm ou 40 mm). A amplitude de movimento, os desfechos relatados pelo paciente e as variáveis radiográficas (morfologia pré-operatória da glenoide, incisura escapular e instabilidade do úmero) foram avaliados.

**Resultados** Todas as medidas de amplitude de movimento, à exceção da rotação interna, melhoraram de forma significativa entre o período pré-operatório e pós-operatório nas duas coortes. Não houve diferenças significativas na amplitude de movimento pós-operatória, pontuação da *American Shoulder and Elbow Surgeons Standardized Shoulder Assessment Form* (ASES) ou escala visual analógica (EVA) de dor entre as duas coortes. De modo geral, a elevação anterior melhorou para 134° ± 16° na coorte de 36 mm e 133° ± 14° na coorte de 40 mm ( *p* = 0,47). A rotação externa melhorou para 37° ± 13° em pacientes da coorte de 36 mm e 35° ± 19° em pacientes da coorte de 40 mm ( *p* = 0,58). A rotação interna aumentou 1,3 níveis vertebrais na coorte de 36 mm e 2,3 níveis vertebrais na coorte de 40 mm. No último acompanhamento, a coorte de 36 mm apresentou EVA de 2 ± 2, ASES de 66 ± 19 e pontuação do *Simple Shoulder Test* (SST) de 6 ± 3. Da mesma forma, a coorte de 40 mm teve EVA de 2 ± 3, ASES de 77 ± 28 e SST de 9 ± 3.

**Conclusões** A ATR de ombro causa melhoras sustentadas da amplitude de movimento e função articular independentemente do tamanho da glenosfera.

**Nível de Evidência** III.

## Introdução

A artroplastia total reversa (ATR) do ombro é um tratamento eficaz para várias artropatias desta articulação e proporciona melhora substanciais na dor e na função do ombro. 1 2 3 Na última década, o número anual de ATRs de ombro mais que triplicou nos Estados Unidos devido à rápida expansão das indicações aprovadas. 4 5 6 7 8 9 10 11 12 13 Embora a ATR de ombro tenda a ser clinicamente eficaz, ainda há dúvidas sobre como diferentes tipos e tamanhos de implantes influenciam os desfechos. Especificamente, a escolha do tamanho da glenosfera é uma das poucas variáveis controláveis pelos cirurgiões.

Há relatos conflitantes sobre o impacto do tamanho da glenosfera sobre os desfechos funcionais após a ATR de ombro. Mollon et al. 14 e outros autores sugeriram que as glenosferas maiores podem ser correlacionadas a melhores desfechos funcionais e radiográficos. 15 16 17 Por outro lado, Schoch et al. e outros autores encontraram evidências insuficientes em apoio à ideia de que o tamanho da glenosfera influencia os desfechos funcionais de forma significativa. 18 19 Sabesan et al. 20 também relataram a dificuldade de atribuir diferenças nos desfechos funcionais a qualquer variável devido à complexidade de controle das variações demográficas e anatômicas na população de pacientes, especificamente em relação ao posicionamento consistente da glenosfera. Além disso, embora a escolha do tamanho da glenosfera seja uma das poucas variáveis controláveis pelo cirurgião, não há diretrizes estabelecidas para determinar as dimensões ideais para cada paciente. Sabe-se que o tamanho da glenosfera geralmente depende da preferência do cirurgião, já que a inserção de uma glenosfera menor é mais fácil; no entanto, o uso de uma glenosfera maior pode reduzir o risco de deslocamento futuro.

O objetivo deste estudo é contribuir para a literatura existente e avaliar a influência do tamanho da glenosfera nos desfechos funcionais da ATR de ombro no acompanhamento em médio a longo prazo. Nossa hipótese é que não há diferença significativa entre os tamanhos da glenosfera em relação à função e dor do ombro.

## Métodos

## População de Pacientes

Este estudo foi aprovado pelo comitê de ética da instituição. O registro institucional de artroplastia de ombro foi consultado usando o código da *Current Procedural Terminology* (CPT) 23472 para identificação dos pacientes submetidos à ATR de ombro. Cada prontuário foi rastreado e os pacientes foram incluídos se tivessem sido acompanhados por um período mínimo de 2,0 anos. Os pacientes foram excluídos caso submetidos à ATR anatômica, hemiartroplastia ou se o tempo de acompanhamento fosse inferior a 2,0 anos. Informações demográficas, como idade à cirurgia, sexo, revisão cirúrgica e índice de massa corporal (IMC), foram registradas. A sobrevida do implante foi definida pela ausência de necessidade de revisão cirúrgica dos ombros após a primeira ATR. O tamanho do implante foi determinado no período intraoperatório com base na avaliação anatômica. Não houve utilização de *software* de planejamento pré-operatório para determinação do tamanho da glenosfera antes da cirurgia.

## Avaliação Clínica

Os desfechos clínicos medidos neste estudo foram as pontuações de amplitude de movimento e os resultados relatados pelo paciente. As pontuações da amplitude de movimento correspondem aos valores de elevação anterior, rotação externa e rotação interna do ombro determinados pelo cirurgião nas consultas realizadas antes e após o procedimento. Os resultados relatados pelo paciente são as pontuações de autoavaliação Escala Visual Analógica (EVA), *American Shoulder and Elbow Surgeons Standardized Shoulder Assessment Form* (ASES) e *Simple Shoulder Test* (SST) com suas respectivas métricas de conversão. A rotação interna foi categorizada como definido por Amroodi et al. 21 O tempo de sobrevida do implante nos pacientes, em que a falência corresponde à revisão ou remoção da glenosfera, foi determinado pela análise de sobrevida de Kaplan-Meier. Quaisquer diferenças nas distribuições de sobrevida foram determinadas pelo teste de log-rank.

## Avaliação Radiográfica

A análise radiográfica foi realizada por dois cirurgiões ortopédicos experientes (BOP e PJC). Um colega (SM) estava à disposição para revisão de quaisquer discrepâncias entre os dois profissionais. A morfologia pré-operatória da glenoide foi analisada segundo o sistema de classificação de Walch. 22 A radiotransparência do úmero e a instabilidade da glenoide foram definidas pelo sistema descrito por Sanchez et al. 23 ; as linhas radiotransparentes acima de 2 mm foram registradas por zona. A incisura escapular também foi avaliada.

## Análise Estatística

As variáveis categóricas foram analisadas por teste de χ2 ou teste exato de Fisher. A normalidade das variáveis contínuas foi determinada por um teste de Kolmogorov-Smirnov e, a partir disso, o teste U de Mann-Whitney ou *t* de Student foi executado. Os resultados foram expressos como média ± desvio-padrão. A amplitude de movimento (ADM) pré-operatória, a ADM pós-operatória e a mudança entre as ADMs pré e pós-operatórias, os desfechos relatados pelo paciente e as medidas radiográficas foram comparados por teste de análise de variância (ANOVA) unidirecional. O coeficiente de Pearson comparou a associação entre o IMC geral e a idade do paciente à cirurgia, ADM e desfechos relatados pelo paciente. O valor de *p* < 0,05 foi considerado significativo.

## Resultados

## Gerais

Cinquenta ombros atenderam aos critérios de inclusão e foram avaliados neste estudo. Glenosferas de 36 mm ( *n* = 38) e 40 mm ( *n* = 12) foram utilizadas. Trinta e quatro dos 38 pacientes com glenosferas de 36 mm eram do sexo feminino, enquanto apenas dois dentre 12 pacientes da coorte de 40 mm eram do sexo feminino ( *p* < 0,01). Catorze homens e 36 mulheres participaram do estudo. Houve 40 casos de primeira ATR de ombro e 10 casos de revisão do procedimento índice.

## Coorte de Glenosfera de 36 mm

A média de idade à cirurgia foi de 72,1 ± 7,4 anos e o tempo médio de acompanhamento foi de 6,7 ± 3,3 anos. Dos 38 pacientes, 34 eram do sexo feminino (89%) e quatro, do sexo masculino (11%). O IMC médio dos pacientes à cirurgia foi de 28,4 ± 5,9 e seu escore médio da *American Society of Anaesthesiologists* (ASA) foi de 2,3 ± 0,6. As três indicações mais comuns para cirurgia foram artropatia ( *n* = 16), falência da hemiartroplastia por fratura ( *n* = 10) e fratura-luxação crônica com rotura do manguito rotador ( *n* = 3). A ATR com transferência do tendão do grande dorsal foi realizada em 13 ombros.

## Coorte de Glenosfera de 40 mm

A média de idade à cirurgia foi de 74,1 ± 7,2 anos e o tempo médio de acompanhamento foi de 4,8 ± 3,1 anos. Dez dos 12 pacientes eram do sexo masculino. O IMC médio dos pacientes à cirurgia foi de 27,3 ± 4,5 e seu escore ASA médio foi de 2,5 ± 0,5. As três indicações mais comuns para cirurgia foram artropatia ( *n* = 6), falência da hemiartroplastia por fratura ( *n* = 4) e fratura-luxação crônica com rotura do manguito rotador ( *n* = 2). A ATR reversa com transferência do tendão do grande dorsal foi realizada em dois ombros.

## Desfechos: Coorte de 36 mm

Todas as medidas de amplitude de movimento apresentaram melhoras significativas entre o pré-operatório e o pós-operatório. De modo geral, a elevação anterior melhorou de 81° ± 46° no pré-operatório para 134° ± 16° no pós-operatório ( *p* < 0,01). A rotação externa melhorou de 26° ± 31° no pré-operatório para 37° ± 13° ( *p* < 0,01) e a rotação interna melhorou 1,3 níveis vertebrais ( *p* = 0,35). Melhoras significativas também foram observadas em cada índice de desfecho relatado pelo paciente. Os escores ASES melhoraram de 33 ± 16 no pré-operatório para 66 ± 19 no pós-operatório ( *p* < 0,01). Os escores SST melhoraram de 2 ± 2 no pré-operatório para 6 ± 3 ( *p* < 0,01). As pontuações pré-operatórias de EVA de dor passaram de 6 ± 3 para 2 ± 2 no período pós-operatório ( *p* < 0,01) ( Tabela 1 ).

**Tabela 1:** Comparação dos desfechos pós-operatórios

Medida	36 mm	40 mm	Valor de p
Elevação anterior ( ^o^ )	134 ± 16	133 ± 14	0,47
Rotação externa ( ^o^ )	37 ± 13	35 ± 19	0,58
Rotação interna	1,3	2,3	0,84
ASES	66 ± 19	77 ± 28	0,05
SST	6 ± 3	9 ± 3	<0,01
EVA	2 ± 2	2 ± 3	0,68

ASES, American Shoulder and Elbow Surgeons Standardized Shoulder Assessment Form; SST, Simple Shoulder Test; EVA, Escala Visual Analógica.

## Desfechos: Coorte de 40 mm

Todas as medidas de amplitude de movimento apresentaram melhoras significativas entre o pré-operatório e o pós-operatório. De modo geral, a elevação anterior melhorou de 74° ± 7° no pré-operatório para 133° ± 29° no pós-operatório ( *p* = 0,02). A rotação externa melhorou de 11° ± 28° no pré-operatório para 35° ± 19° ( *p* = 0,04) e a rotação interna melhorou, em média, 2,3 níveis vertebrais ( *p* = 0,28). Melhoras significativas também foram observadas em cada índice de desfecho relatado pelo paciente. Os escores ASES melhoraram de 30 ± 19 no pré-operatório para 77 ± 28 no pós-operatório ( *p* < 0,01). Os escores SST melhoraram de 3 ± 4 no pré-operatório para 9 ± 3 ( *p* < 0,01). As pontuações pré-operatórias de EVA de dor passaram de 7 ± 3 para 2 ± 3 no período pós-operatório ( *p* < 0,01) ( Tabela 1 ).

## Comparação dos Desfechos da Coorte

Não houve diferença significativa na amplitude de movimento entre as duas coortes. Quanto aos resultados relatados pelo paciente, a coorte de 40 mm apresentou pontuação SST significativamente maior, mas não houve diferença nas pontuações ASES ou EVA. A coorte de 36 mm teve quatro complicações submetidas à revisão cirúrgica, enquanto a coorte de 40 mm não apresentou nenhuma. Veja uma comparação completa dos desfechos pós-operatórios na Tabela 1 .

## Radiografia

Havia radiografias pré-operatórias de 30 ombros. A classificação da glenoide de Walch pôde ser feita em 18 ombros da coorte de 36 mm: A1 ( *n* = 12), A2 ( *n* = 3), B2 ( *n* = 1), B3 ( *n* = 1) e D ( *n* = 1) e 12 ombros da coorte de 40 mm: A1 ( *n* = 4), A2 ( *n* = 3), B2 ( *n* = 3), B3 ( *n* = 1) e D ( *n* = 1). A morfologia pré-ATR não pôde ser avaliada em três ombros devido à fratura anterior de glenoide ( *n* = 2) e ATR anatômica prévia ( *n* = 1). Havia radiografias pós-operatórias de todos os pacientes. A incisura escapular pós-operatória foi observada em oito dos 38 pacientes da coorte de 36 mm e dois dos 12 pacientes da coorte de 40 mm no último acompanhamento ( *p* = 0,46). A instabilidade da glenoide foi observada em três ombros da coorte de 36 mm. A instabilidade do úmero foi observada em seis ombros da coorte de 36 mm e três da coorte de 40 mm. A reabsorção da tuberosidade foi observada em seis ombros da coorte de 36 mm.

## Complicações e Revisões

Quatro complicações foram revistas cirurgicamente na coorte de 36 mm. Um paciente submetido à primeira ATR foi revisto 2 meses após a cirurgia devido ao desacoplamento da glenosfera. Um paciente submetido à primeira ATR apresentou afrouxamento da placa de base 5,8 anos após o procedimento, que foi revisto. Um paciente submetido à primeira ATR foi submetido à revisão 8,9 anos após a cirurgia devido a uma infecção. Em um paciente, a prótese foi explantada por causa de uma infecção 3,8 anos após a ATR. A coorte de 40 mm não apresentou complicações ou revisões.

## Discussão

Há diversos estudos acerca do papel do tamanho da glenosfera nos desfechos da ATR de ombro. 14 16 17 24 Embora haja alguma literatura sugerindo que glenosferas de tamanhos maiores estão associadas a melhores desfechos, outros estudos sugerem que não há vantagens em tal abordagem. Aqui, observamos a ausência de diferença significativa na amplitude de movimento ou nos desfechos relatados pelos pacientes que receberam glenosferas de 36 mm ou 40 mm.

Pesquisas prévias sugeriram que o tamanho maior da glenosfera estava associado à maior rotação externa pós-operatória. 14 15 16 Haidamous et al. observaram que pacientes com glenosferas maiores apresentavam probabilidade significativamente maior de apresentar rotação externa acima de 30 ^o^ . ^16^ Da mesma forma, Mueller et al. demonstraram que, em 5 anos de acompanhamento, os pacientes com glenosfera de 44 mm tiveram uma média de 12° a mais de rotação externa do que aqueles com glenosferas de 36 mm. 14 Um estudo biomecânico com cadáveres, feito por Langohr et al., 19 também demonstrou que o aumento do tamanho da glenosfera não elevou a rotação externa. Assim, há achados conflitantes acerca da relação entre o tamanho da glenosfera e a rotação externa. Em nosso estudo, não observamos diferença significativa na rotação externa pós-operatória entre as glenosferas de 36 mm e 40 mm (36 mm: 37°; 40 mm: 35°; *p* = 0,58). No entanto, nossos achados podem ser decorrentes da amostra muito menor de pacientes com glenosferas de 40 mm. Nossa hipótese é que, se houvesse um aumento no número de pacientes com glenosferas de 40 mm, esses indivíduos apresentariam rotação externa significativamente maior. Em um estudo futuro, seria valioso controlar as variáveis de confusão, como idade do paciente, nível de atividade e indicação cirúrgica, ao avaliar a relação entre o tamanho da glenosfera e a rotação externa.

As pontuações de desfechos relatados pelo paciente têm grande valor na avaliação da função do ombro após a ATR. Em um estudo com 370 glenosferas de 38 mm e 219 glenosferas de 42 mm, Schoch et al. não observaram diferença significativa nos escores ASES e Constant em um acompanhamento médio de 2,6 anos. 18 Da mesma forma, Sabesan et al. 20 também demonstraram que o tamanho maior da glenosfera não confere vantagem em relação aos desfechos relatados pelo paciente. Em nosso estudo, não observamos diferenças significativas nos escores ASES ou SST entre as coortes de 36 e 40 mm em uma média de 5 a 7 anos após a cirurgia. No entanto, houve uma diferença significativa nos escores SST (36 mm: 6 ± 3; 40 mm; 9 ± 3; *p* < 0,01). Mais notavelmente, ambas as coortes relataram uma pontuação EVA média idêntica, de 2, o que sugere que todos os tamanhos de glenosferas são eficazes na redução da dor em candidatos à ATR. Esses achados são promissores já que, independentemente do tamanho da glenosfera, os pacientes podem obter desfechos excelentes com a ATR.

Múltiplos estudos investigaram o papel do tamanho da glenosfera na incisura escapular em pacientes submetidos à ATR. 14 17 24 Embora alguns estudos sugiram que a glenosfera maior reduza o risco de incisura escapular, outros estudos relatam que essa vantagem não existe. 14 15 17 24 Em um estudo de acompanhamento de médio prazo, Mueller et al. não encontraram diferenças significativas na incidência de incisura escapular em pacientes com glenosferas de 36 mm ou 44 mm. 14 Similarmente, Mollon et al. 17 relataram que 10% da coorte de 38 mm e 9,5% da coorte de 42 mm apresentavam incisura escapular no último acompanhamento. Em um estudo controlado randomizado, Torrens et al. observaram que 49% dos pacientes com glenosferas de 38 mm tinham incisura escapular, enquanto apenas 12% dos pacientes com 42 mm a apresentavam. 24 Condizente com os estudos retrospectivos anteriores, descobrimos que não houve diferença significativa na incisura escapular com base no tamanho da glenosfera. Vinte e um por cento da coorte de 38 mm e 17% da coorte de 40 mm apresentaram incisura escapular no acompanhamento final ( *p* = 0,49). No entanto, mais pesquisas prospectivas são necessárias antes da obtenção de conclusões definitivas sobre o papel do tamanho da glenosfera na incisura escapular. Os estudos que sugerem que não há diferença eram de porte maior, mas também retrospectivos em comparação ao estudo randomizado prospectivo menor, que relatou que glenosferas menores aumentam o risco de incisura escapular.

Este estudo tem limitações. O pequeno número de pacientes que receberam glenosferas de 40 mm dificultou a obtenção de conclusões definitivas entre as duas coortes. A ausência de imagens pré-operatórias de alguns pacientes reduziu a capacidade de entender as características prévias desses indivíduos. A natureza retrospectiva deste estudo cria um viés inerente nos resultados. Alguns pacientes submetidos à ATR em nossa instituição faleceram ou foram perdidos ao acompanhamento, o que pode ter alterado os resultados.

## Conclusão

Este estudo contribui para a literatura acerca da relação entre o tamanho da glenosfera e os desfechos clínicos. Descobrimos que não houve diferenças significativas na amplitude de movimento, desfechos relatados pelo paciente ou incisura escapular entre as coortes de 36 mm e 40 mm. Os dois tamanhos de glenosfera proporcionaram alívio da dor em longo prazo aos pacientes. É importante ressaltar que não houve complicações ou revisões quando a glenosfera maior, de 40 mm, foi usada.
